# Infection prevention and control in neonatal units: An ethnographic study of social and clinical interactions among healthcare providers and mothers in Ghana

**DOI:** 10.1371/journal.pone.0283647

**Published:** 2023-07-07

**Authors:** Gifty Sunkwa-Mills, Kodjo Senah, Britt Pinkowski Tersbøl

**Affiliations:** 1 Ghana Health Service, Central Region, Kasoa, Ghana; 2 Global Health Section, Department of Public Health, University of Copenhagen, Copenhagen, Denmark; 3 Department of Sociology, University of Ghana, Accra, Ghana; Agha Khan University, UNITED REPUBLIC OF TANZANIA

## Abstract

**Introduction:**

Healthcare-associated infections (HAIs) are a global health challenge, particularly in low- and middle-income countries (LMICs). Infection prevention and control (IPC) remains an important strategy for preventing HAIs and improving the quality of care in hospital wards. The social environment and interactions in hospital wards are important in the quest to improve IPC. This study explored care practices and the interactions between healthcare providers and mothers in the neonatal intensive care units (NICU) in two Ghanaian hospitals and discusses the relevance for IPC.

**Methodology:**

This study draws on data from an ethnographic study using in-depth interviews, focus group discussions involving 43 healthcare providers and 72 mothers, and participant observations in the wards between September 2017 and June 2019. The qualitative data were analysed thematically using NVivo 12 to facilitate coding.

**Findings:**

Mothers of hospitalized babies faced various challenges in coping with the hospital environment. Mothers received sparse information about their babies’ medical conditions and felt intimidated in the contact with providers. Mothers strategically positioned themselves as learners, guardians, and peers to enable them to navigate the clinical and social environment of the wards. Mothers feared that persistent requests for information might result in their being labelled “difficult mothers” or might impact the care provided to their babies. Healthcare providers also shifted between various positionings as professionals, caregivers, and gatekeepers, with the tendency to exercise power and maintain control over activities on the ward.

**Conclusion:**

The socio-cultural environment of the wards, with the patterns of interaction and power, reduces priority to IPC as a form of care. Effective promotion and maintenance of hygiene practices require cooperation, and that healthcare providers and mothers find common grounds from which to leverage mutual support and respect, and through this enhance care for mothers and babies, and develop stronger motivation for promoting IPC.

## Introduction

Healthcare-associated infections (HAIs) remain a global health challenge [1, 2], with associated direct and indirect costs to health institutions, families, and individuals [3, 4]. Neonatal intensive care units (NICUs), with neonates receiving complex medical therapy in a highly technical environment, are challenging environments in which to maintain patient safety [5]. HAIs are responsible for more than a quarter of the estimated neonatal deaths in hospitals in LMICs [6]. In Ghana, the overall HAI prevalence rate is 8.2% among hospitalized patients [7].

In the NICU, mothers of babies on admission are important stakeholders, and their involvement is critical in improving the quality of care [8, 9]. Although mothers are not solely responsible for the care of their babies, their constant presence in the therapeutic space renders them important stakeholders in care, whose concerns and roles need to be considered [8, 9]. This also requires that the underlying social relations of power are recognized and considered [10]. The medical encounter has been portrayed as a place where patients are subordinated to physicians’ domination. The unequal power relationships between healthcare providers (HPs) and clients (including patients, caretakers, and mothers) are a central factor at the core of addressing quality of care [11–15]. The differences in provider and client access to power and decision-making are further accentuated by the different statuses of providers and clients [16]. In Ghana, research has shown how power relationships affect the quality of care women receive during childbirth [17, 18]. HPs play a key role in involving and empowering mothers. However, mothers’ reliance on the perceived expertise of HPs enforces unequal power relations [12, 19].

The joint endeavour of meaningful collaboration between HPs and mothers in managing the risk of infection in this context is complex and compounded with challenges [20–22]. In this context, HPs are often more focused on the provision of clinical care and are uncertain about how to engage parents and relatives in care delivery [8, 9]. Although IPC as a form of care may seem less of a priority to HPs, management of the risk of infection constitutes a crucial aspect of care.

Limited research exists on the social environments of NICUs in low- and middle-income settings including the interaction between HPs and mothers [23–26]. Using Positioning Theory, this ethnographic study explores care practices in two NICU wards in Ghana, to identify challenges and opportunities for improved IPC.

### Conceptual framework

Positioning Theory is a psycho-sociological concept of how people position themselves and others within society and in institutions [27–29]. It is concerned with revealing the patterns of reasoning that underlie how people behave toward one another [28]. This theory has been applied to workplace interactions in fields ranging from public relations [30, 31] to interprofessional relations in healthcare, including how HPs see themselves in relation to other colleagues, patients and their relatives [32, 33]. Harré and colleagues explain that "positioning theory studies refer to cognitive processes that are instrumental in supporting the actions people undertake, particularly by fixing for this moment and this situation what these actions mean" [28].

HPs orient themselves to the hierarchies and duties attached to their professional functions in the hospital setting. Communication and negotiations about hygiene and IPC compliance also take place in this context [32]. Among HPs, collaboration across organisational boundaries remains challenging, and power dynamics affect the strategic choices about how and with whom to collaborate [13]. Positioning theory [28, 30, 34] is employed to shed light on the necessity and functionality of positions in this context.

Positioning theory has been used to examine how people produce and explain their behaviour and that of others, and how positions are invoked and negotiated [29, 34–37]. Positioning and other-positioning may result in marginalization, decreased opportunities, and exclusion [38]. HPs are continuously engaged with mothers in the NICU context, with its characteristic structural and socio-cultural working conditions. Focusing on the positionings of HPs and mothers, the relevant factors and the framework within which care is delivered are explored.

From the Foucauldian perspective, the hospital ward can be described as a ‘heterotopia’, a relatively segregated place in which several spatial arrangements and rules co-exist, practices and power structures interconnect, and various lines of interest, identity, authority, and activity intersect [39]. Doctors, nurses, administrators, patients, and families, who are involved in this space subscribe to a set of cultural norms and base their expectations and decisions on professional information, knowledge, and background [40–42].

Power shapes social inequalities experienced by individuals and communities as well as health collaboration, participation, and ownership [43]. In hospital settings, where there is an asymmetrical power difference between clients and HPs [11, 44], any form of collaboration toward improving the quality of care is associated with complexities. Continuous attention should be focused on the care practices in such contexts [45]. Using positioning theory, we explore the potential to attend to and strengthen care practices in hospital wards.

## Methodology

### Study setting

Ghana is a West African country with a population of about 30.3 million and is divided into 16 regions, constituting the northern, middle, and southern zones [46]. Ghana has 10 regional-level hospitals which form secondary-level referral points from primary care centres, and 5 teaching hospitals providing tertiary-level care in the public sector [47]. This study occurred in two purposively selected hospitals in southern Ghana: the Greater Accra region and the Eastern region. The Greater Accra region was selected because it is the national capital and has some of the largest health facilities in the country. The Eastern region was selected due to logistical reasons, with its proximity to the national capital. This study was conducted in the NICU of a tertiary-level hospital and a secondary-level hospital, which were purposively selected as part of a larger field study on HAIs in Ghana [7, 47]. The two hospitals selected for this study have an average HAI prevalence rate of 10.2%, which is above the overall HAI prevalence rate of 8.2% among hospitalized patients in Ghana [7].

The tertiary-level hospital (hereafter referred to as Facility A or FA) is a 2,000-bed hospital in Accra in the Greater Accra region and serves as a referral centre for most hospitals in the southern zone and beyond. FA has a 55-bed NICU and a 261-bed maternity unit. The NICU admits approximately 2400 neonates yearly. The secondary-level hospital (hereafter, FB) is in Koforidua in the Eastern region and has a 356-bed capacity that serves the population of the Eastern and other nearby regions. FB has a 30-bed NICU and a 54-bed maternity unit. The NICU admits about 1000 neonates yearly.

### Study design

We used an ethnographic approach involving qualitative in-depth interviews, focus group discussions (FGDs), and participant observations to collect data between September 2017 and June 2019. Ethnographic research emphasizes "being there" and gaining an insider perspective by observing and interacting with people in the setting, as participants become more comfortable with the researchers’ presence [48]. Ethnographic studies require long periods in the field to experience the everyday lives of participants [49, 50]. This can provide a deeper insight into social phenomena, and help in understanding the organisational and cultural aspects of patient safety research [51].

Multiple data collection methods were employed as the hallmark of a good qualitative study [52, 53] and to present an in-depth understanding of the topic under study. FGDs help to gain an understanding of how individuals collectively construct meanings and provide deeper and richer data due to group dynamics [43]. Participant observation was done to familiarize with the care processes and appreciate the relationships and interactions between the various participants. The first author (GSM) conducted most of the in-depth interviews and FGDs, with the help of two trained research assistants, who have degrees in health-related fields and experience in qualitative research.

The first author (GSM) is a female medical doctor and Ph.D. researcher with a background in anthropology and public health. GSM, under the guidance of the Ph.D. supervisors, BPT (last author, an associate professor of public health with a background in anthropology and qualitative research), and KS (second author, a professor of social science with decades of experience in qualitative research) trained the research assistants and also supervised them during data collection. The researchers were not familiar with the participants before the study.

### Recruitment and data collection

Purposive sampling was used to recruit HPs working in the two hospitals. We considered the various categories of HPs on the wards during the selection, to achieve diversity in terms of staff cadre and level of experience. HPs were approached during their break period, informed about the research, and invited to participate. The study included doctors, nurses, auxiliary nurses, midwives, hospital managers, IPC coordinators, and ward in-charges at the maternal and NICU wards with more than 6 months of experience in the hospital. The study excluded HPs working in the outpatient departments and those who were on study leave or transfer at the time of the study. Forty-three HPs participated in in-depth interviews.

Women 15 years and older, whose babies had been admitted to the NICU for a minimum of 48 hours were eligible to participate in the study. The mothers were selected purposively to ensure that they had spent different periods in the NICU so they could share their varied perspectives on care. Mothers were recruited from the maternity and NICU wards, as some mothers spent their time between the two wards. Mothers were approached, informed about the study, and invited to participate. None of the participants who were invited refused to participate in the study. A total of 32 mothers participated in the in-depth interviews, and a convenience sample of 40 eligible mothers participated in 6 FGDs, 3 in each hospital, with 6–8 women per group.

Interviews lasted 45 to 60 minutes and FGDs lasted 60 to 90 minutes. Interviews were conducted face-to-face in the hospital, and in quiet side rooms on the wards, or in available conference rooms or meeting rooms. Demographic information was collected. A semi‐structured interview guide (S1 Appendix) which had been pilot-tested was used to capture participants’ experiences with ward interactions and IPC compliance, but it was open to include other perspectives. Some mothers’ interviews were conducted in English and others in Twi.

At the point of data saturation, no new information was generated from the interviews. We conducted 6 FGDs with 40 mothers. We considered this sample size sufficient to fulfil the objectives of this study, based on a predicted thematic saturation after 5 FGDs, with an allowance for an extra FGD after data saturation.

Participants were provided refreshments (drinks and snacks) during the interviews.

The first author and 2 research assistants conducted participant observations intermittently in the two hospitals. The observations were done on the wards during both the day and night shifts, using an observation guide (S2 Appendix). This was done on 2 or more days in a week in each hospital over the period of the research. The combination of participant observation and interviews provided insight into how perceptions were translated into action [52–54]. During participant observations, researchers participated in activities, assisted by handing over items during procedures, and supported HPs when they needed help to fetch items or to arrange the wards. Informal conversations were held with HPs during work or while they were on break. We took down observation notes and documented any interesting incidents during the observation period. Observation notes were taken during participant observation (S3 File).

### Data analysis

Interviews were audio-recorded and transcribed verbatim. Interviews conducted in Twi were translated into the English language during transcription and then checked for accuracy. Data were analysed thematically based on the objectives of the study [55, 56]. Relevant contextual information from interview notes and field notes were incorporated for further ethnographic analyses [50]. The transcripts were uploaded to QSR N Vivo 12 to support coding and analysis. The data was triangulated, and similar codes were grouped into categories. Initial codes were descriptive and close to the data [57]. The categories were then regrouped into subthemes and themes. Our theoretical orientation was drawn from the positioning theory [28, 34] which informed the framework for analysis. The initial reading of the transcripts was done by all authors. GSM conducted the majority of the analysis; however, the co-authors (KS and BPT) read and coded several interviews. During data analysis the developing themes were also discussed with the co-authors and other researchers including conflicting perspectives which represent the complexity of social life and interactions on the ward. The second author (KS) was engaged to resolve any discrepancies. To maintain rigor in the analysis, we aimed for reflexivity by having a research team with diverse backgrounds. Team discussions were held to incorporate diverse perspectives while interpreting the findings to maintain objectivity in the research. We used the COREQ checklist (Tong et al., 2007) [58] to report our research as it is the standard guide for reporting qualitative research studies.

### Ethical approval and considerations

Ethics approval for this study was obtained from the Ethical Review Committee (GHS-ERC 07/03/2017) of the Ghana Health Service. Written and verbal information and explanation about the research objectives were given to participants before each interview. The interviewees all gave written informed consent by signing a consent form after they had been informed about the study and assured of confidentiality and anonymity. Pseudonyms were used to ensure the anonymity of participants.

## Findings

HPs were aged between 20 and 59 years and worked in various capacities in the hospital (Table 1). The mothers who participated in this study were representative of postnatal mothers in the selected wards (Table 2).

**Table 1:** Characteristics of healthcare providers who participated in the study (n = 43).

Demographic characteristics	Number	(%)
**Age**		
20–30	22	51
30–39	18	42
>40	3	7
**Marital Status**		
Single	14	33
Married/ Partner	28	65
Divorced	1	2
**Staff cadre**		
Manager	4	9
Doctor	8	19
IPC Coordinator	2	5
Nurse/Midwife	24	56
Other	5	12
**Years in current position**		
< 5	35	81
6–10	7	16
> 10	1	2
**Location/ Service level**		
Tertiary (FA)	18	42
Regional (FB)	25	58
**Gender**		
Male	10	23
Female	33	77

**Table 2:** Characteristics of mothers who participated in the study (n = 72).

Demographic characteristics	Number		(%)
**Age**			
15–19	4		6
20–29	29		40
30–39	25		35
40–49	14		19
**Marital Status**			
Single/Other	25		35
Married	47		65
**Education**			
None	10		14
Primary	26		36
Secondary	19		27
Tertiary	14		19
Postgraduate	3		4
**Number of days on admission**			
<14		51	71
15–28		17	24
>28		4	5

### Navigating the NICU

The NICUs of the two hospitals had many similarities in terms of organization of care, procedures, and routines. The number of HPs on the wards changed constantly due to the shift system, regular reshuffling, and the presence of medical and nursing students on rotations and internships.

In FA, the NICU consists of three cubicles. Babies are admitted to cubicle 1 when they are severely ill. Cubicle 1 has more incubators and radiant warmers, and usually has the attention of more nurses as the babies are critically ill. The babies are moved to cubicle 2 when their clinical condition improves, and then to cubicle 3 prior to discharge.

Most mothers move between the maternity ward and the NICU to feed their babies at scheduled times. Other mothers commute from home or a nearby hostel to feed their babies every 2–3 hours.

In FB, the NICU has two cubicles—one for babies born within the hospital and the other for babies referred from external health facilities. Mothers come in to attend to their babies 2–3 hourly, and when specifically requested by HPs. In the initial stages of treatment, babies are placed in open cots, under radiant warmers, or in specialized incubators that monitor their oxygen level, temperature, respiration, and other vital signs. It is common to hear beeping alarms and humming sounds from machines in the NICU environment.

HPs maintain a constant presence in the NICU, overseeing the contact between mothers and babies. Experienced HPs are familiar with the ward setting, routines, and technology; however, mothers often find this space alienating. In FA, mothers sit in compactly arranged plastic chairs in an open space in the middle of each cubicle when they come in to care for their babies. In FB, mothers sit by their babies’ cots to care for and breastfeed them. Outside the scheduled hours for contact with their babies, mothers often remain in the background trying to understand what is going on while keeping an eye on their babies from a distance. In trying to cope with this situation and find their place as part of the ward community, mothers positioned themselves as learners, guardians, and peers.

#### Mothers’ position as learners

Mothers consistently mentioned that they were neither introduced to the routines of the ward nor their roles in the care of their babies. A young mother who had been referred to FA from a smaller hospital narrated how she waited all night to be attended to by a doctor.

*“It was a little bit frustrating because the ambulance brought me in around 10:30 pm…. on Thursday evening … and by the time I got a bed it was 4:00 am on Friday. You can imagine! There’s no chair to sit on, nowhere to lie. The ambulance had left. …they took me to the emergency… then they left me hanging till they were ready for me (MT1)*

After she had delivered through a caesarean section, her baby was transferred to the NICU and was kept in a cot near the nurses’ station until a doctor was available to conduct an assessment of the baby. Her husband did the initial follow-up on the baby’s condition, and she only saw her baby a few days later, while recovering from surgery. She lamented that she received little information about the condition and progress of her baby. More than half of the mothers interviewed shared similar experiences reflecting poor communication and ensuing feelings of marginalization.

About a third of the mothers mentioned that they were uncertain about who would address their concerns. These mothers also mentioned that they felt uncomfortable asking questions about their babies.

*“What will I ask? I am neither a doctor nor a nurse. I do not understand what is going on”*.

*(MT10)*

Struggling with information deprivation, mothers said they felt relieved when they received information about their baby’s treatment or progress.

*“I remember one of the doctors…he came to tell me the following day…that my baby was alright. And I thanked him for the information he brought to me, but I still didn’t know where exactly they had sent my baby.” (MT9)*

Mothers were anxious about the lack of bonding with their babies through skin-to-skin closeness. Mothers with critically ill babies had to wait longer to embrace their babies, who were often attached to tubes and machines. Mothers became fast ‘learners’, as they observed and tried to understand routines and activities in care and how HPs handled the babies. Mothers negotiated with nurses to be allowed to do more, such as changing diapers and bathing their baby as the baby’s medical condition improved. Mothers took these initiatives with careful consideration of the potential negative reactions of HPs, to avoid being reproached for engaging with their babies without permission to do so.

In all FGDs, mothers mentioned that they were grateful and relieved whenever HPs offered guidance on how to care for their babies. They appreciated it when HPs encouraged and empowered them to participate in caring for their babies. Mothers underlined the importance of HPs’ support in helping them to understand the invasive procedures, changes in weight, and other concerning aspects of the baby’s condition. Mothers acknowledged that although it was a stressful time, support and feedback from HPs could help them have a more positive experience.

*“I take a lot of interest in what is going on. I ask a lot of questions…I get very interactive with the nurses when they are not busy…if they are busy, I don’t stress them… I can imagine their frustrations … I mean if they can’t answer my questions at the time, they would get back to me later.” (MT17)*

Although there seemed to be no clear role for mothers in many scenarios, they made themselves available to learn and perform any tasks related to the care of their babies. They would typically ask a lot of questions, which met a wide range of responses from the nurses, sometimes favourable, and other times discouraging.

*“When they come around to do their work, I ask them questions. …. Some smile and talk to me, others don’t.” (MT18)*

During the FGDs, mothers made comments such as:

*“They do not even exercise patience to listen … when we ask questions, they do not take their time to explain things well to us.” (MT45)*

*“Sometimes they come and tell you that today, your baby will be under the light, but they do not explain why. Is the light supposed to help the baby improve? Is there something wrong? They do not say anything to us.” (MT52)*

#### Mothers’ position as guardians

Mothers generally perceived the hospital as a stressful environment. Some mothers speculated about the potential risk of infection. They mentioned the inadequate provisions available for them and often complained that the time allotted for them to be with their babies was inadequate. A mother mentioned that the associated anxiety made her so stressed that she often could not sleep well at night.

Mothers longed to be fully informed about their baby’s progress and have opportunities to raise questions about care, especially when they perceived their babies to be at risk. A mother indicated that she did not fully trust HPs with the care of her babies, due to previous experiences.

*“I find myself being nosy… I would like to know when they are administering the medication… when I come, I want to follow up on it… please, did you give this medication at this time to my baby? There have been more than four occasions when it hasn’t been administered… because we are many, and probably… they forgot… but once you remind them, they do it.” (MT1)*

Mothers reported how they sought to watch their babies closely whenever they had the opportunity so that they could detect any issues with their baby’s condition and bring it to the attention of HPs.

A mother mentioned that at one point, she alerted a nurse about her baby’s intravenous line that had come off, but was dissatisfied with the apathetic response she received.

When mothers felt that HPs were not keeping up with the babies’ needs, they worried that it would affect the baby’s condition. This, they said, was especially so during the night duties, when there were fewer doctors and nurses on duty.

*“While some mothers at the ward have their babies close by, mine is lying so far away. I cannot leave him there and go to sleep, so I sit by his bedside and sleep in the chair if I feel tired”. (MT20)*

If permitted to do so, mothers assumed some of the nursing tasks, once their babies were stable. Nurses would correct mothers to have procedures and tasks done according to how things, in their view, should be done. Mothers who adapted, felt better accepted by the HPs. Thus, mothers are directed to be ‘good mothers’. Mothers who do not adjust or who questioned or confronted HPs on the informal or formal rules of the ward risk being labelled as ‘difficult’ by HPs.

In FA, a mother expressed concern about the risk of infection due to the close arrangement of cots and incubators, which also left little room to perform care activities or have any privacy.

*“I think they can have another place for us the mothers to breastfeed instead of being in the same room with the incubators and other machines… the place is small*, *and the mothers are many… we can even spread infection to the babies”*. (MT9)

Mothers also paid attention to spaces reserved for medical procedures. HPs took laboratory samples from the babies and performed other procedures on a designated table with a linen cover. Mothers sometimes complained that the linen cover on the table was not changed frequently, thereby posing a risk of infection. A mother also complained that she had noticed ants in her baby’s cot and expressed worry about the dilapidated state of the mattress.

As guardians, mothers paid attention to hygiene in the NICU. A mother said:

*“I just had my own way of extra sanitizing my hands. I pick the chair with my elbow because I don’t want to infect my hands so that I don’t defeat the purpose.” (MT32)*

#### Mothers’ position as peers

Mothers in the NICU did not have direct access to any official structured guidance or support systems provided by the hospital. Collective instructions, orientation, and support were not routine procedures on the wards, so mothers generally received sparse instructions about how to act and cope on the ward during this stressful time. Instead, important information, instruction, and advice were provided by other mothers, a form of improvised and unofficial peer education in the NICU. Mothers discretely established networks with peers and offered advice right from the first days in the NICU to help others find their way in the opaque rules and norms of the wards.

Mothers described watching other mothers closely and that this interaction helped them in attempting to become more “competent” in the gaze of HPs. Sharing experiences with other NICU mothers was an important source of emotional support. Mothers created and facilitated an informal platform to share experiences. Mothers who had stayed longer in the NICU often became unofficial teachers, with the new incoming mothers literally calling themselves “students.” The “students” typically observed what their peer mentors were doing and took cues from their approaches.

*“For me, I have not started breastfeeding my baby, but sometimes when I see people breastfeeding their babies, I observe” (MT37)*

Peer support also reminded mothers of routine hand hygiene practices as the experienced mothers were already used to this. The mothers relied heavily on the support and advice generated by this association. When mothers were not informed about the purpose of specific medical procedures or equipment that their baby was exposed to, they relied on more experienced mothers who could provide comfort by giving them some insights:

*“When I came and noticed my baby had been put under the light for phototherapy, I was scared … I was confused and was crying until another mother whose baby had previously been put under the light called me, and explained to me that it was going to help my baby. She assured me that my baby was going to be ok” (MT30)*

Some mothers also depended on the support and encouragement from family members and friends to help them during the period of hospitalization. Although relatives came around to support them, there were visiting restrictions that prevented them from having access to the NICU. HPs explained that they had concerns about too many relatives coming in, as it would increase the risk of infection for the babies. Some mothers were however displeased with the restrictions as it left them feeling even more lonely and excluded.

#### Positionings of healthcare providers

In the following, we describe the ways in which HPs positioned themselves.

#### Healthcare providers as professionals

The major professionals in the hospital wards include doctors, nurses, and other clinical and non-clinical staff. Both hospitals have an IPC coordinator—a nurse with the task of supporting, communicating, and facilitating hygiene procedures in the wards. The IPC coordinator worked across professional groups and tasks. In terms of responsibility for implementing HAI prevention strategies, HPs maintained that it should be done by everyone including clinical staff, non-clinical staff, and mothers.

As professionals, HPs placed themselves in a position as knowledgeable and powerholders, legitimated by their educational and professional qualifications and the hierarchical structures of the hospital. HPs felt the need to make their authority known and tangible to mothers and established boundaries to control the daily routines on the ward. One HP said:

*“Sometimes you’re busy trying to set a line for a baby, only for a mother to tap you on the shoulder trying to find out what is wrong with their baby; it can be really distracting… but it’s ok, because sometimes we also need them to be around to give us information about the babies”(D7)*

These boundaries were not only of a clinical nature but focused on controlling social interaction, to maintain order in interactions with mothers and their relatives. Sometimes, when convenient for HPs, they would disregard these boundaries and allow mothers or family members into the ward outside the designated visiting hours to assist in carrying specimens to the laboratory or purchasing medicines from the pharmacy.

Although communication with mothers was often done in an authoritarian way, there were also instances of kind and empathetic communication. HPs would encourage mothers to take care of their health and advise on when and what to eat to have a good flow of breastmilk. HPs focused on clinically authorized standards, levels, and measurables, for example, they expected mothers to sometimes express and measure breastmilk for babies who had to take specified amounts at a time. Mothers were expected to relate to these clinical objectives and remain cooperative, even though this was sometimes challenging for some mothers.

#### Healthcare professionals as caregivers

The position of the clinical caregiver was central in the NICU. HPs conducted physical examination, set intravenous lines, suctioned and resuscitated babies among many other tasks, and they monitored babies in critical conditions and performed other routine tasks in the NICU. HPs also provided other forms of care including feeding, cleaning babies, changing diapers, and assisting mothers who attempted to breastfeed their babies:

*“It is her first time having a baby, and sometimes even handling the baby is a problem … the idea that her baby is in NICU, the anxiety is already high, so even touching or holding the baby is another problem; so, she will ask you to do it for her. You have to encourage them and let them know that they are babies, and they will go home with them so bit by bit … they are able to grasp everything you say, and as time goes on, you will see them positioning the baby to the breast and breastfeeding them” (N15)*

HPs admitted that they did not always have enough time to explain things to mothers, and they did not always prioritize it; the busy workload, large number of babies, and rapid patient turnover were described as overwhelming. The night shifts were perceived to be more demanding.

*“The night shift is more tedious… and there are limited staff too. …And because the mothers do not come up to breastfeed during the night, the workload is increased… you have to do everything by yourself” (N14)*

We noted from our observations that the night shifts usually involved fewer administrative tasks and social activities, but nurses had to do more work, as there were fewer nurses assigned to care for the same number of babies during the night. Senior consultants did not work on night shifts and had to be contacted on the phone if there were critical issues or a baby was in a critical condition and the doctor on duty needed direction.

HPs described their challenges in coping with the demands of caring for severely ill babies while attempting to address the physical and emotional needs of mothers. Therefore, they mostly focussed on the babies, despite the opportunities to address mothers’ worries and engage mothers in a participatory role in care.

All HPs associated some aspects of their work as caregivers with a risk of infection, given the regular exposure to body fluids and ‘unseen’ microorganisms.

#### Healthcare providers as gatekeepers

HPs determined the extent to which mothers could participate in the care of their babies. For extremely ill babies, nurses were observed guiding the mother to hold or hand over materials during procedures. Nurses, however, positioned themselves as ‘gatekeepers’ by restricting the amount of time mothers had direct physical contact with the delicate preterms, and explained that this was to ensure the baby’s safety. Mothers’ participation in caring for babies was particularly limited during these periods. When mothers were allowed to hold their babies and carry out different care tasks, HPs felt the need to control how mothers handled their babies due to their vulnerability.

A nurse mentioned that mothers were sometimes inattentive or slow to respond to instructions, and this increased the work of HPs as they had to supervise such mothers to ensure timely responses to instructions such as purchasing drugs or following up on laboratory specimen results, in the interest of the baby’s health. During our observations and interactions, one doctor described a practice of ‘covering’ babies by administering antibiotics as ‘prophylaxis’ to babies who were perceived to be at risk of infection from a clinical perspective, or from the environment when they are paired with other babies in the same cot when the NICU was full.

When discussing IPC, HPs highlighted that mothers need to maintain a minimum level of hand hygiene—with emphasis on washing hands at the entrance of the NICU, and after changing the baby’s diapers. HPs directed mothers to follow instructions through posters, e.g., proper handwashing, not handling of mobile phones while breastfeeding, not picking things from the floor, etc. Mothers were often instructed ’not to touch’. One mother said:

*"Over here you don’t take something for someone. You can’t take anything for someone, even if it’s a cot sheet… even if you want something, you have to tell the nurse, especially with the cups that we express the milk into. They take the cup themselves, you don’t have to touch it" (M21).*

HPs worked within an environment with both human resource and supply constraints. One HP noted:

*“… ermmm sometimes the workload alone overwhelms you, so I won’t say you tend to forget your hand hygiene, but it comes as if it’s a burden or something of that sort.” (N28)*

HPs were expected to maintain clinical standards with limited equipment and materials. Cots, phototherapy machines, radiant warmers, and apnoea monitors were available but not in sufficient numbers for the care of neonates admitted. Various professionals sometimes competed for resources, and glove use was monitored by ward matrons and used sparingly.

In gatekeeping, HPs controlled resources available to mothers too. For example, limited quantities of hand towels were kept at the entrance where mothers wash their hands, and liquid soap was disbursed in diluted quantities in bottles as a strategy to minimize wastage.

Some HPs in FA expressed concerns about overcrowding in the NICU as it made it difficult to monitor mothers adequately, especially during feeding times. HPs mentioned that they expect mothers to cooperate with them as they the HPs oversee the health needs of their babies. A nurse indicated that ‘difficult mothers’ do not comply with nurses’ instructions.

*“Err… Ideally, there should be a nurse at the entrance during the time that the mothers come, to supervise them to wash their hands at the proper place when they enter and when they are leaving… but for some time now … we do not have a permanent public health nurse who will assist with those things… so one of the nurses speaks to the mothers at the gate …so they wash their hands before they enter the cubicle, but when they are going most of them don’t wash their hands.” (N3)*

This nurse here also indicated that instruction to mothers regarding IPC is ideally the role of a public health nurse, rather than the responsibility of all staff.

### Collaboration to improve hand hygiene

HPs in this study appreciated and discussed the relationship between HAIs and poor adherence to IPC protocols, especially hand hygiene. One doctor mentioned that HAI is not projected enough among HPs, and that more evidence must be demonstrated to convince HPs of the significant impact of hand hygiene on preventing HAIs. One HP admitted to neglecting hand hygiene protocols in circumstances that require unusual responses such as emergencies where time is of essence.

HPs mentioned that the frequent lack of materials for hand hygiene makes IPC compliance challenging, especially in FA where the handwashing sinks in the cubicles were often out of order or had no water flowing.

*“Yeah, sometimes you might come and there wouldn’t be soap so you would have to go and get some from somewhere else, and also sometimes it is not practical for me… for example, if I’m going to examine like 15 to 20 babies, I can’t wash my hands after everyone.” (D1)*

HPs indicated that they needed more commitment from those at the management level to help address gaps in IPC on the wards.

*“There is no allocation of funds for IPC to the best of my knowledge… so anytime you request for funds… then you hear the complaints of no funds …there is no money." (N23)*

Although HPs might be called for IPC training, IPC-related materials were routinely lacking:

*"For instance, when we went for the workshop, they taught us a lot of things, but when we come to the ward, we don’t get the items. For instance, when decontaminating or washing bedsheets you need to wear utility gloves. They will say it, but it’s not available." (N18).*

Although HPs did not actively engage mothers in conversations on hand hygiene or infection control, mothers who took the initiative to follow protocols were often encouraged by the HPs.

*“Some mothers were also very cautious to the extent that if they pick a chair with their hand, you see them rubbing their hands with the sanitizer before they touch the baby. It was impressive. It was very good.” (N6)*

During observations, HPs were seen monitoring each other and commenting on who was observing IPC protocols or performing hand hygiene appropriately. One HP said:

*“When I come on a shift and a staff is performing a delivery without an apron, I would quickly go for an apron and come and put it on for her.” (N41).*

HPs also considered it important to support and look out for each other:

*“If you see a colleague who has taken off the gloves and the hands are very soiled… and he or she just wants to move to the next thing by just sanitizing, you prompt them “you can see your hand is visibly soiled, so what do you”? Then the person will say “handwashing”, and the person goes to wash their hands. So, like everybody is prompting someone and we are all on the same page.” (N18)*

Discussing the challenges of IPC implementation, a HP commented on the difficulty in reaching management with suggestions of improvement due to ’lines of command’.

*“The challenges have to do with the structure… if I identify something that I think can help improve IPC*, *I have to communicate that to somebody…the head of the department… who will then have to discuss it at the management meeting… and once I’m not there to articulate and explain what it is*, *the person’s interest will influence the outcome of this idea… so if he doesn’t see the need for what you are saying*, *what it means is that your idea is already aborted prematurely*.*”*(N22)

During interactions with one HP who was also a manager, we asked about how sustainability of an IPC intervention could be secured. He promptly responded that we should “forget about sustainability” and added that even if we "just provide the gloves, cleaning agents, and other IPC supplies" for a few months, some babies would still benefit, even if it is not sustainable. The HP continued to describe his own efforts to secure running water in the ward, ’chasing the engineers…spending hours looking for them.’ He added that they eventually came and fixed the pipes, but two days later, there was no running water. "I don’t have the time to chase engineers", the HP concluded.

## Discussion

This study used an ethnographic qualitative methodology to assess IPC efforts in the clinical and social environment of two NICUs in Ghana. Inspired by the positioning theory, we explored how healthcare providers and mothers positioned themselves opposite each other in the social field of the hospital and navigated their roles in the hospital setting. We focused on key aspects of the positionings of HPs and mothers, and how this helps us understand the social environment and the "positioning" of IPC in this setting. Research participants make sense of their experiences and actions and also justify their positions and practices by reference to other people, processes, and hospital structures surrounding them in the ward environment.

### Mothers’ positioning as a form of agency

The hospital and its organizational and staff structure is an arena of asymmetrical roles and power dynamics [59, 60]. HPs have a high status due to their education and position in the hospital, while mothers are expected to be compliant with the priorities of clinical management. These asymmetrical power dynamics lead to the strategic positioning of the HPs and mothers. The interactions between HPs and mothers are of great importance in improving the quality of clinical decision-making and ultimately maternal and new-born health outcomes [26, 61–63]. Researchers have demonstrated a need to support bonding between mothers and their babies through early and repeated physical contact [64, 65]. Mothers experience distress when restricted from physical contact with their babies. Early separation from the mothers is also a significant stressor for neonates, influencing emotional and cognitive development with possible long-term health consequences [66]. Similar to our findings, mothers have reported feelings of neglect and lack of supportive treatment in hospital wards in other settings [67]. Other studies have reported that powerful HPs can frustrate less powerful mothers through neglect or withholding required services [60, 68].

Parental presence in NICU, together with support and education by nursing staff reduces the stress that parents experience [69]. Implementing initiatives in this direction in low-income settings to improve the level of satisfaction with care would demand resources and a shift of the culture and practices shaping social interaction and positioning on the ward [61, 67].

In our data, mothers positioning as guardians was fuelled by lack of information and lack of trust in the dedication of HPs to ensure the best possible care. As guardians, mothers’ agency was limited. They could watch their baby if they were allowed in the ward, but they could not interfere with the clinical care. Notification about a drip that had fallen out or other unintended clinical events not addressed by HPs, would possibly spur irritation from HPs.

However, the mothers exercised agency in other ways: positioning themselves as compliant learners aimed to pave the way for goodwill, information and up-dates from HPs. As ‘good mothers’, they avoided interfering with the routines of HP and they could carefully time their request for information, attention to a drip out of place, or being granted more time next to their babies’ incubators.

Mothers also exercised agency as they teamed up and offered or received peer guidance. Mothers demonstrated their emic insight and capacity in their support to new mothers. This way, the experienced mothers inherently also supported the HPs’ routines on the ward. Peer networks have shown themselves to be beneficial in care, and helpful in allaying the concerns and experiences of marginalization in terms of clinical care and hygiene [70–72].

### HPs’ positioning as a form of self-protection

As HPs positioned themselves as professionals and gate keepers, they continuously refer to a need to maintain control and power over the presence and actions of mothers and relatives, so as to be able to focus on clinical tasks and prevent unforeseen events that would steal time. HPs justify this with reference to the supreme priority of clinical tasks. This tendency is compounded by the fact that work at the NICU is challenged by a patient-load above ward capacity and staff numbers that do not match the patient-load. One HP described how contributing to innovation is difficult because the distance to decision-makers where suggestions could be heard and discussed is too great, which indicates that HPs sometimes feel disempowered.

HPs communicated with authority, provided limited information on conditions of the neonates and sought to maintain a focus on clinical care and outcomes, similar to findings in other studies [73, 74]. Although HPs had opportunities to engage mothers to partner with them in caring for the babies and in promoting IPC, the stressful situations in the NICU did not encourage an interactive and collaborative approach, and there is a possibility that HPs were concerned that this would affect their authority. Other studies have reported that HPs felt that engaging with families created additional demands on their time [75, 76]. However, non-clinical care, such as communication with and support to parents and relatives has been shown to influence perceptions of the quality of care [77, 78].

One HP mentioned the practice of "covering babies"- providing preventive treatment with antibiotics to babies who were perceived to be at risk of infection from overcrowding or the general ward environment- which can be viewed as "an extension of infection prevention and control measures". Willis and Chandler have raised concerns about treating infections as a quick fix, rather than addressing the underlying factors of poor hygiene among others. They further noted that antibiotics have become a ‘quick fix’ in fractured health systems, being used as a substitute for hygiene in resource-constrained settings [79]. Labi et al argue for continuous surveillance of infections in the NICU to guide antibiotic treatment guidelines and improve neonatal morbidity and mortality [80]. The positioning of HPs could be described as a response to organisational ‘injustice’ [81] where HPs feel exploited and disenfranchised from influences in their workplace, as shown in our findings. Arnold et al noted that HPs in Afghan hospitals were frustrated by the injustices they experienced as a result of their own powerlessness within the healthcare system [82]. Aberese-Ako et al [81] emphasise the need to develop health care organisations that are able to produce people-centred care and continuous quality improvement. Our data points to this need as well. Organisations that cannot protect and care for their own people, can hardly provide quality care for clients. In principle, IPC constitutes a form of mutual care—a care practice that should be prioritized to keep babies, mothers, and HPs safe.

### Strengths and limitations

The study involved long durations of immersion in the hospital wards to mitigate observation biases and using interviews to gain a better understanding of the topic under exploration. This strengthens our understanding of how positioning intersects with the HPs’ and mothers’ experiences in the hospital context and how this affects care delivery. The inclusion of mothers’ perspectives further strengthens the findings of this study.

Some mothers were interviewed in Twi. It is possible that there may have been translational variability, and that nuances in the language could have been missed in the process of interviewing, transcription and analysis. To mitigate this, researchers carefully considered the approach to phrasing interview questions to facilitate easy comprehension. Researchers were also skilled in interviewing and established a good rapport with participants which made the sharing of information easier. Transcripts were checked against the original audio files to ensure the nuances of the local language had been well captured, to limit translational variability.

Questions to participants were not always phrased or probed the same way. The findings are therefore informed by the experiences of the participants and the researchers. However, researchers were reflexive, and there were constant discussions between researchers to ensure that the objectives of the study were understood.

## Conclusion

This study shed light on issues in the socio-cultural environment of the wards which reduce priority to IPC. The research demonstrates the need for HPs and mothers to reflect on their positionings in relation to each other, to promote better communication and collaboration. These positionings have provided an analytical construct by which we capture patterns of interaction and power. While Harré et al. speak of "fixing for this moment the meaning of actions" [28], we consider the positionings expressed in our data to be of a more lasting nature. This is because the organisational and societal context that co-create these positions are more or less unchanging and the stressors and dilemmas of both HPs and mothers present on the ward, are therefore continuously reproduced.

The WHO global report on infection prevention and control [83] indicates that IPC interventions can reduce HAI rates HAIs by 35–70%. There is however a need to put in more effort to ensure that IPC is placed at the centre of hospital care alongside clinical care, in sustainable ways, with full-time professionals dedicated to IPC, allocated IPC budget, surveillance, and a suitable patient-staff ratio. While human resources and other resources and materials for IPC are important, the social environment and interactions in hospital wards are also fundamentally important. To improve IPC in a sustainable way at the health facility level, an enabling environment must be created, as well as an institutional culture that considers important socio-cultural processes that impact care practices including IPC-related care.

Effective promotion and maintenance of hygiene practices requires cooperation between HPs and mothers, and the need to find common grounds from which to leverage mutual support and develop a stronger motivation for preventing HAIs.

This requires a shift in emphasis from the biomedical focus to collaborative practice in the appropriate socio-cultural context, with a facilitating environment that also supports mothers’ confidence as stakeholders. A better understanding of mothers’ experiences helps identify strategies to involve them as partners in care delivery. Further research into strategic approaches towards a partnership to improve the quality of care would be beneficial in ensuring that mothers receive quality care for themselves and their babies.

In addition to training and capacity building for HPs, the organisational culture in hospitals should prioritise IPC as a form of care for both HPs and clients. IPC knowledge, skills, and tools should be accessible to both HPs and clients to facilitate best practices. Withholding knowledge or skills as a way of maintaining power, respect, or superiority [82] should be discouraged. Rather, HPs should be empowered through an enabling environment to maximise the provision of quality care.

This study explores the positionings of HPs and mothers and focused less on actual cases of HAIs. An important next step would be to link positioning, perspectives, and practices to the occurrence of HAIs.

## Supporting information

S1 Checklist COREQ (COnsolidated criteria for REporting Qualitative research) checklist. (PDF)

S1 Appendix Interview questions. (DOCX)

S2 Appendix Participant observation guide. (DOCX)

S1 File Quotes reflecting mothers positioning. (DOCX)

S2 File Quotes reflecting HPs positioning. (DOC)

S3 File Participant observation notes. (DOCX)

## List of abbreviations

FA: Facility A (Tertiary Hospital
FB: Facility B (Secondary Hospital
FGD: Focus Group Discussions
GHS: Ghana Health Service
HAIs: Healthcare-Associated Infections
HPs: Healthcare providers
IDIs: In-depth interviews
IPC: Infection Prevention and Control
LMICS: Low-and middle-income countries
MOH: Ministry of Health
NICU: Neonatal Intensive Care Unit
WHO: World Health Organization
