# Supplementary material for: Infection prevention and control in neonatal units: An ethnographic study of social and clinical interactions among healthcare providers and mothers in Ghana
Source: PLoS One. 2023 Jul 7;18(7):e0283647. doi: 10.1371/journal.pone.0283647 (PMC10328309; doi:10.1371/journal.pone.0283647)
Supplement: S1 Appendix — (DOCX) [file pone.0283647.s002.docx]

| Box 1  **Sample of interview questions- Mothers**  Tell us about what brought you and your baby to NICU?    •When you arrived at NICU, how were you received?  How did the health workers talk with you about your baby?  •What did the health workers explain about your baby’s condition?  •What did the health workers communicate to you subsequently about the progress of your child?  •How do the health workers communicate with you?  Did you have to assist in taking care of your child sometimes? Please describe your experience    •In what other ways do you want to be involved in the care of your baby  Share with us any precautions you take when handling your child  How were the health workers were helpful?  •To what extent are your concerns addressed by HWs?  Do you feel the health workers were helpful?  Do you feel they were acting professional? (e.g. did they put on gloves to handle your child etc.- explore what mothers perceive as "professional")  Do you sometimes see the health workers wash their hands?  •Can you explain why they wash hands during their work or procedures?  What action would you take if you see a health worker touching your baby without washing their hands?  Can you describe the state of the environment where your child was admitted;  •Did you have any concerns?  What support do you receive from: Family members/Friends/ Peers or other mothers in the NICU/Health workers  Do you have any questions concerning your child? | Box 2  **Sample of interview questions- Healthcare providers**  Can you tell me about the nature of your work as a staff of this unit?  We would like to talk with you a little bit about infection prevention and management. Do you think healthcare-associated infections are a problem in this hospital? Can you explain?  What infection prevention and management activities do you carry out in your own day-to-day work?  In your opinion, who has responsibility for the management of healthcare-associated infections in your hospital? Why?  Are you aware of any specific standards associated with infection prevention and management?  In your view, what are the main challenges for preventing and managing healthcare-associated infections in your hospital?  How do you feel about encouraging your colleagues to comply with infection control practices?  What role do you think patients and their caregivers have to play in reducing HCAIs?  Do you feel anything would need to change to reduce the risk of healthcare-associated infections? If so, what are these changes?  Are there any questions that you would like to ask? |
| --- | --- |
