# Supplementary material for: Infection prevention and control in neonatal units: An ethnographic study of social and clinical interactions among healthcare providers and mothers in Ghana
Source: PLoS One. 2023 Jul 7;18(7):e0283647. doi: 10.1371/journal.pone.0283647 (PMC10328309; doi:10.1371/journal.pone.0283647)
Supplement: S2 Appendix — (DOCX) [file pone.0283647.s003.docx]

| Box 1  **Observation Guide for Health workers, non-clinical staff, Mothers/ Caregivers, Patients**  Describe the person’s roles and responsibilities on the ward  Describe any protective gear worn by the person to perform general duties (gloves, masks, boots, aprons, goggles, gown)  Describe observed hand hygiene practices: Is it done frequently?  Describe how the person reacts to/lack of reaction to ‘dirt’ (‘matter out of place’) in the environment  What seems to be important?  Any leadership role in the environment/ role of authority  Anything that influences actions of person  Any protocols being followed /Any checklist available for reference  Any peculiar oversights  Note any other action of interest performed by person  Note any other observations of interaction, communication, or general environment  Any leadership role in the environment/ role of authority | Box 2  **Observation Guide for Places/ Procedure spaces**  Describe Physical Appearance of Place including general cleanliness. Any first impressions?  Presence –who/what is there -or not there? e.g., furniture, windows  Note any peculiar smell  Are there noises or disruptions from the immediate environment or nearby?  Note availability or absence of Waste Bins in Offices and Client Service areas  Observe distribution of Sharps Disposal Boxes  What appears missing?  What values does the place promote or discourage? / Where do values conflict?  How does the place and space influence social actions? e.g., breastfeeding? / Place for changing diapers etc.?  Is the environment conducive to ensure privacy for purpose?  Describe the positions and distribution of IPC equipment  What PPES are worn for procedures ?  Observe any surfaces which are used for procedures. Describe state of cleanliness  Observe disposal of waste materials and sharps after procedures  Note removal of PPEs and hand hygiene if performed after procedures |
| --- | --- |
