# Supplementary material for: Infection prevention and control in neonatal units: An ethnographic study of social and clinical interactions among healthcare providers and mothers in Ghana
Source: PLoS One. 2023 Jul 7;18(7):e0283647. doi: 10.1371/journal.pone.0283647 (PMC10328309; doi:10.1371/journal.pone.0283647)
Supplement: S1 File — (DOCX) [file pone.0283647.s004.docx]

**Supplementary Table S1. Mother Positionings**

| **Positioning: LEARNERS** |  | **Quotes** |
| --- | --- | --- |
| **Sub Theme** |  |  |
| Initial learning experience and surprises  Introduction to the NICU  Need for more baby time  Sparse guidance and directions  Support from Nurses  Bonding with the baby |  | *It was a shock especially when I came and I realized that my baby had been put in a respiratory machine, I thought that was the end of the story.*  *Hmm, you see for me since I have not given birth before so I can’t tell which is right and which isn’t. I don’t really know how a baby should be catered for).*  *“I wanted to ask, but I asked the sisters (nurses) here that, if one wants to know what is wrong with her child what is she supposed to do? And she told me to ask those people who have things around their necks who are the doctors and they are the ones who can tell me know what the problem is with my child. And I said okay so this morning when I came I saw one of them inside there and I asked what the problem with my child was”.*  *“I was asked to hold on with breast milk, and that my baby is…. they were telling me he’s suffering from convulsion. I’m not a nurse, I am not a doctor …. but convulsion?”*  *Okay we came… they made my husband take over everything and they said I can only come and see the baby at 9 am. So when I arrived, I mentioned my baby’s name and asked where he is, and they showed me … that I should go in. so I went and saw the baby… I carried him and prayed for him*  *“Yes, that’s what I have to do, if I had my way, I’d ask them to allow us to spend all the time with our babies. If we were given the chance to stay here from morning till the next day, as for me I’ll do that but I don’t think it is possible”.*  *“Yes, because if you see how small he is, I’m not allowed to carry him. The nurses bring him out and put him on their laps but for me when he is inside, I can stretch and hold his arm but I don’t carry him”.*  *The security man, the security woman basically is the boss at the gate, so she basically decides the time that we come in… that is the time that we are called to enter.*  *Sometimes … we come early and sit down for a while… then they will tell us to come inside. If we come inside too, they don’t allow us to sit down with the babies for long.*  *All that they say, there is a paper there… that wash your hand, but as to how to wash your hands. There is no instruction or pictorial thing anywhere uhuh. So, everyone does what they want because they are supposed to, wash their hands and clean them and then come*  *The nurses here are good. Because if anything they take their time to explain… they can even discuss things with us, and they take care of the children.*  *Some nurses carry and take care of the baby nicely; you will even think it’s their own baby. But with others they just feed and leave the child there and leave even if the baby is crying and the mother is not around; she won’t mind the baby*  *“I don’t know how to hold him, so one of the nurses taught me”*  *“When we came they told me that you shouldn’t be worried too much about the weight because when she came she was… it was like she was developing jaundice so they put under the light. They say normally it draws their energy a bit by the time you realize their weight will go down. So it went down, I think on Sunday doctor Edem weight her and she was 1.18 kg. Yesterday Auntie Jane weight her again and she was 1.19 kg”.*  *The baby is tied to your chest so you can talk to the baby, you can sing, and you can chat with the baby for 1 hour.* |
| Participative learning  Taking interest in care-giving activities  Encouragement to overcome fear |  | *They taught as how to carry the babies, care for them, how to feed them and because they are small, how best to handle them in order for him to recover and get the necessary strength.*  *I take a lot of interest in what is going on. I ask a lot of questions…I get very interactive with the nurses when they are not busy…If they are busy, I don’t stress them… I can imagine their frustrations trying to get a baby’s vein to get blood… I mean if they can’t answer my questions at that time, they would get back to me later.*  *There is one mother … I think they told her that she will breastfeed the baby herself and she was like… she is scared; so one of the nurses encouraged her- that it’s your baby- you carried her 9 months in your womb so what are you scared about? I don’t know what she was scared about. So, she did that and the mother was okay.* |
| Not knowing what to ask or who to ask  IPC care with limited resources |  | *What will I ask? I am neither a doctor nor a nurse. I do not understand what is going on*  *They are the ones who have to tell us this or that. If they do not, how will we know?*  *Ermmm the problem also here is that everybody so in scrubs, so differentiating who is who is a bit difficult…The only difference might be the stethoscope*  *As for this place. How the nurses and doctors are; I am afraid because of the way some of them shout on some of us… but there are some of them I can ask, because of the way they relate to us -it’s fine.*  *The one I met in the morning is not the same person I met in the afternoon so I don’t know anyone. I’m afraid that if I go and ask they will shout at me that I am coming to teach them their job or something*  *Sometimes when you are asking questions, they get angry… those on duty*  *In the morning there were towels, but this afternoon, when I came, there was no towel… I used my handkerchief.*  *It’s not always that there is a towel. I shake my hands to make it dry but sometimes it doesn’t work so I just use my dress to clean it.* |
| Need for information  Awaiting information  Receiving IPC information  Receiving education on self-care |  | “*Oh when I came… my husband went in, I asked him what is wrong with the baby; what did they say? He said they told him the baby was not due for delivery when I gave birth to him, so his breathing is not good; so they have placed a drip… oxygen on him. When I came inside and I saw it, I asked the doctor there… he told me that because he is not breathing well that is why he is being given the oxygen and that it will support him- so that is when I understood…”*  *“My husband usually pressures me to ask questions anytime I feel like, because me, I thought when you come to NICU and you’re identified as the mother of the baby, the nurses or doctors should call you and tell you what is wrong with child so that you as a mother you’d also know. But since I’ve come nobody has told me exactly what the problem is. So I was saying to myself that, when I leave and I’m coming back, I’d ask one of them what exactly the problem is. Or in case I see that doctor around, I’d kindly ask him what is wrong with my child”*  *“All the lab results we brought… I don’t know the results- I don’t know what it is saying; I don’t know because when you bring the report they ask you to put it down- nobody is saying anything”*  *“They said maybe some people have illnesses or dirt on their hands, so you have to wash your hands before you come inside to take care of the baby”.*  *They said before I enter, I should wash my hands and that when I finish feeding the child and I am going I should also wash my hands, and that when I go I should be taking in soup and iced kenkey… I should express a lot of milk for both of them (twin babies) in order for them to get energy*  *E7: So when coming, take off your cap. If you go to the ward then you wear it because at the ward they will tell you to put it on because you are a patient. As for that one you can’t prevent them but when coming here, you should at least take it off so that even after washing your hands you would not bring different things here. It is a nice way of talking to us so that the person will understand why you are telling her to remove the white thing when coming. Because the white thing is on her head, she will go and bath with it. She will go to the wash room with it. She will sleep with it.*  *Even may be she is sitting down chatting and there is a (inaudible), she will put her head there. You don’t know what is on the (inaudible). There are certain things I think if the mothers were being told, it will help all of us so that most of them will be okay.* |
| Learners being sidelined in care  Receiving varying information |  | *There is this nurse… if you talk to her or ask her any question, she won’t mind you… so if I have a problem I just ask someone else”.*  *Sometimes they tell us that when we finish feeding, we can change the diapers. Sometimes the nurses tell us to change it. Some too tell us not to, and that they will do it by themselves.*  *I think if a doctor should take care of a child he should finish with the child because if they keep changing the doctors, each one will come with a different view… If today they ask you to do this test another doctor will come and say that he doesn’t want this test … so this really worries us.*  *Yes. And the mothers too they complain that anytime they come here, they stay for too long. Maybe she has been around for more than 2 weeks and the baby has been doing several labs but they’ve still not told her anything. So in this kind of situation, I think that you the doctor shouldn’t wait for the person to ask…they are scared to ask the doctor about what is wrong with their child.*  *So, anytime we come, they should inform us on what is going on, so that we know what to do. Sometimes too you can see that the baby looks healthy but still he/she is lying there, sometimes they’d say there is a medicine they need to inject for maybe 10 to 14 days, the 14 days will pass by and still the child will be lying there.*  *. Right now when you ask someone she will say her child has been discharged but another doctor comes in and says no the child shouldn’t be discharged. So the baby should sleep there some more, you see, so these things will make the mothers talk about it.*  *“They should say it in the morning, in the afternoon and also in the evening because there are some people, in the morning they don’t come only in the afternoon. And there is someone too who hasn’t been here before, and some too when you see them and you question them, they only say they are here to see their babies, then just pass without washing their hands. So I’m stressing on the fact, always when we come, the nurses should talk it about, every time, regardless of the number of times they have already said it. So that everyone will be used to it because as the days pass by, new people keep coming in”* |
| ***GUARDIANS*** |  |  |
| Keeping a constant physical presence in the wards |  | *I am always with them … when they ask me for something, I do it for them.*  *Like labs and all those things… and they ask me to buy medicines*  *I sit there… the veranda… and when I am hungry I go and eat then I come back*  *They can call you to come for a paper and go and do labs …so, if you sit there and they need you they can come and call you …*  *… no one has told me anything… but I see them come and call some mothers to take papers to go for medicine and do labs. So, I think that if we go far it won’t be good … maybe they might need a drug to inject the baby, and if you are not around…* |
| Looking out when there are few staff at night |  | *I think if they can do it 1-2 patients to a nurse, or 3 patients to a nurse, it will be better ... you can see that a whole cubicle has only 2 nurses …. If your child is crying and another child is crying, sometimes she attends to that one and forgets the other one… or forget that she has not given this one the medication or feed. It happened to me last time… I put the breast milk down and I said okay when you finish … feed my child for me. Since 9am, I came around 12… they had not fed him* |
| Monitoring the babies for visible signs of danger |  | *I touch him and then I hold him up and then I check everything, whether everything is correct. …* ***…*** *This morning … I realized that where they set the line, the baby’s hand was swollen; very big. So, I just drew their attention that the baby’s hand is swollen.*  *I told them so when I was leaving, they were removing it. I check my baby for everything…* *I check”* |
| Being alert  Being aware of risks |  | *At night, I cannot even sleep. When I doze off and I hear a baby crying, I quickly wake up and go and check on my daughter*  *“How can I even sleep? While some mothers at the ward have their babies close by, mine is lying so far away. I cannot leave him there and go to sleep so I sit by his bedside and sleep in the chair if I feel tired”.*  *If the doctor saw one baby with an infection… and the other baby is not having that same infection- If he doesn’t change his gloves, the other baby can get the same infection* |
| Watching baby’s progress |  | *Oh, when I gave birth … I realized that his breathing … how his breathing was… right now it has improved; and they have also removed the oxygen from him. So, I can see for myself that he has improved* |
| Identifying and averting or correcting errors  Alerting the staff of safety hazards  Managing stress and worries |  | *If I am not okay with how they are feeding him, I can tell the nurse that… oh if you let the baby lie down and you feed… he can choke.*  *So, when feeding him they raise him and feed him; when they are done I take him and pat his back.*  *When I got here this morning I saw that my baby’s skin was dry…is it flaking? .. he has a dry skin… so I asked the nurse if I should get Vaseline for him... she said okay*  *Sometimes you just come and express milk and care for the baby, by the time you are leaving you are still worried. Some come and see their babies and they are worried but if you are able to sit them down and explain the condition, it will help reduce their anxiety. If don’t tell them anything, when they go out they are not fine.* |
| Mindful of hygiene  Mindful of environment |  | *I just figured it’s intensive care and I know that for intensive care when you go, you wear gloves …. I just had my own way of extra sanitizing my hands. I pick the chair with my elbow because I don’t want to infect my hand so that I don’t defeat the purpose*  *“I am even afraid of this mattress. Do you have any that I can buy for my baby to replace this? I don’t think I am comfortable with him lying on this mattress*  *… you see the cloth that they sleep on… Sometimes it falls on the ground… then you see the nurse picking it up and using it on the baby*  *…some of the incubators to, when they are cleaning, they use the same bowl; the same rug… cleans inside an incubator, and around it… then when he is done, he goes to a different incubator ... with the same bowl.. the same rug, water. Only God knows what you might have picked from that incubator to the next one.*  *In the hospital here, there are gutters around, and it is not just the hospital… there are people also residing around here so there is refuse and dirty water and other things.*  *The environment, okay, the environment is okay, it’s okay*  *I don’t see any dirt or any rubbish on the floor or anywhere, whenever we use diapers or anything.. gloves and anything we put it in the dustbin. That’s what makes it clean* |
| **PEERS** |  |  |
| Seeking support  Need for patience and empathy |  | *When you come on the first day and ask any questions, there is no one to answer them for you but we sometimes get the mothers who have already been here for a while to educate us on how things work here.*  *I think ermmm some of the nurses they need a little bit patience.*  *Because for some of the mothers, … the condition in which their babies are in right now… ermmm it’s disturbing them. May be you advise the person to do something, but the person might forget because she is thinking about her baby. So the nurses, sometimes they have to be patient with the mothers, talking to them more yeah* |
| Hanging together |  | *… sometimes there are no seats. So, you have to stand, or you may be going round as a mother. Some people are in the hostel. They are fortunate to be in the hostel so when it is break time, they go back to the hostel. For those of us who come and go home daily, we have to hang around when there are no seats… roaming till the time is almost up for you to come back.* |
| Observing other mothers and learning |  | *“For me, I have not started breastfeeding my baby, but sometimes when I see people breastfeeding their babies, I observe”.* |
| Looking out for each other |  | *If you don’t have anyone to do the rounds for you at NICU you will be very sorry… if you don’t have anybody you will be very sorry. A mother giving birth and going through all this stress is not easy.*  *I realized she (mother) left her chair. So this morning when she came I said we don’t do that. …the nurses are really mad at you. Just pray that they have forgotten.*  *I call or look out for them… You have left your chair, you have done this, and you have done that… saying it in a nice way.* |
| Lack of guidance and support by staff  Assurance from peers |  | *When I came and noticed my baby had been put under the light for phototherapy, I was scared …*  *I was confused and was crying until another mother whose baby had previously been put under the light called me and explained to me that it was going to help my baby. She assured me that my baby was going to be ok”.* |
| Need for constant reminders |  | *Even when you’re inside you see that some mothers, when they come to their babies, maybe after changing the diapers, they won’t wash their hands and then she will come and sit down and use the same hands to feed her baby. Meanwhile when you tell her she won’t mind you, unless maybe a nurse sees and tells her. But as for me, I do. And I think … many people come and go every day, so I would ask that the everyday the nurses talk about handwashing.* |
