# Supplementary material for: Infection prevention and control in neonatal units: An ethnographic study of social and clinical interactions among healthcare providers and mothers in Ghana
Source: PLoS One. 2023 Jul 7;18(7):e0283647. doi: 10.1371/journal.pone.0283647 (PMC10328309; doi:10.1371/journal.pone.0283647)
Supplement: S2 File — (DOC) [file pone.0283647.s005.doc]

**Supplementary Table S2. Healthcare provider Positionings**

| **Positioning** | **Quotes** |
| --- | --- |
| **Positioning as Professionals** |  |
| Working under resource constraints | *For instance, when we went for the workshop they taught us a lot of things but when we come to the ward we don’t get the items. For instance, when decontaminating or washing bedsheets you need to wear utility gloves. They will say it but it’s not available.*  *At a point in time, I have about two to three rotation nurses here. So if about ten people should come on duty that day, if we wash the towels they do not dry …if it rains, these towels won’t get dry*  *There are times like that for instance when you come on night duty and all the gloves have been used*  *There is no allocation of funds for IPC to the best of my knowledge… so anytime you request for funds ... then you hear the complaints of no funds …there is no money.* |
| Knowledge acquisition | *But we go for workshops and then, recently, we went for a workshop and then they brought this idea of segregation of waste and hand washing; proper handwashing to prevent infections and then proper use of right tools for dressing. “* |
| Workload | *… ermmm sometimes the workload alone overwhelms you so I won’t say you tend to forget your hand hygiene but it comes as if it’s a burden or something of that sort.* |
| Protecting biomedical standards and norms  **Positioning as Caregivers** | *“… sometimes you need to measure the volume of feed given to a baby, so you ask the mother to express into a cup and not breastfeed directly. If she refuses to do any of these but tells you that, for her, her breastmilk does not flow if the baby does not suckle… then you are just torn between what to do… if she gives it directly from the breast, you cannot measure. (D7)* |
| Risk of infection  Potential risks from relatives  Blaming of relatives as ‘the problem’ | *We do delivery, sometimes we change their diapers,*  *… and relatives too coming in, touching their babies … Sometimes when they come from their various homes, they don’t wash their hands and they come and touch the baby*  *You tell them only one relative is allowed but later they will come in their numbers.*  *Their relatives are the problem. We normally tell them that if they want to touch the patient they have to wash their hands… Some of them feel reluctant (NLW2)*  *Because they are coming from outside… You also don’t know where they are coming form so … ermmm we don’t know what they are carrying into the place but the good thing is that at least, for the fathers when they come in we give them protective clothing (NC2)* |
| Challenges in caregiving at night | *…And imagine a cubicle of like 15-20 babies and you have like one or two staff per cubicle meanwhile the night duty is more tedious* |
| Involving mothers in IPC | *As to how well to involve them in IPC… we haven’t done much by calling them and educating them that when you come here you have to wash your hands before you leave. We don’t actively do that…*  *Their knowledge on IPC measures …most of them do not really know. When they are feeding their babies some of them drop their phones and quickly pick it up… and just use that same hand to feed; they drop their towels, pick it up and hang it on their shoulders… the next moment they put the baby on it and start burping* |
| **Positioning as Gatekeepers** |  |
| Guarding resources/  Reducing IPC effort by mothers | *“**One key thing I have observed is that, you know, we have a hand washing bay, and it has a bold inscription “staff only” … Relatives who come to the ward don’t even have the idea that touching patients or the surroundings can give them infections … So they touch … but when they are going there is no hand washing bay… when they get to the nurses so-called “staff only” bay and they want to wash their hands, then you see staff shouting “no, no no, you are not supposed to wash your hands there” and so they will just have to walk out with the infection”* |
|  |  |
| Perceiving mothers as difficult | *“Hmm, well, at times, we get some difficult mothers who wouldn’t want to go the path that you are treading, no matter how you advise or counsel them; they will do whatever they like... So at times, they get in your way”.* |
| Expectations of a ‘good mother’ | *“ A good mother is somebody who will be willing to discuss with the doctors or the nurses if she has a problem to discuss it and not just go around saying what she feels. And somebody who will also be able to cooperate in the sense that if we want to do lab, if we want to run a test for the baby, we want to do other things, she will accept, understanding the procedure and then accepting that okay go ahead in the best interest of the baby, aha. I think that will be a good mother”.* |
| Monitoring and supervision of mothers | *Err… Ideally there should be a nurse at the entrance during the time that the mothers come, to supervise them to wash their hands at the proper place when they enter and when they are going(leaving)… but for some time now …the one who does that one is mainly a public health nurse but … we don’t have a permanent public health nurse who will assist in those things… so one of the nurse speaks to the mothers at the gate before they enter so they wash their hands before they enter the cubicle but when they are going most of them don’t wash their hands* |
| Socially determined boundaries of gatekeeping  Restricting relatives and enforcing IPC | *Nowadays… It has become part of our policy that during delivery, each pregnant woman should come with one person, ... Why, because we realised that we are not many, the staffs are not many. .. so sometimes when the relatives are here, they help them when it comes to buying something for them, taking something for them and other things.*  *We try to practice infection prevention and control so we do not allow relatives to visit. We make them wash their hands before entering*  *If it’s not preemie (preterm) they do not stay here for a long time we try to practice the infection prevention and control so we do not allow relatives to visit. (NC 5)* |
