# Supplementary material for: Infection prevention and control in neonatal units: An ethnographic study of social and clinical interactions among healthcare providers and mothers in Ghana
Source: PLoS One. 2023 Jul 7;18(7):e0283647. doi: 10.1371/journal.pone.0283647 (PMC10328309; doi:10.1371/journal.pone.0283647)
Supplement: S3 File — (DOCX) [file pone.0283647.s006.docx]

**S3 FILE_SAMPLE OF OBSERVATION NOTES**

| Health Facility code: FA |
| --- |
| Participants: Mothers, Nurses, Doctors |
| Unit name: NICU |
| Observer: GSM |
| Date: 19/10/2017 Observation Time: 9.30-12.30pm; 2.00pm- 5.00pm |

When I walked into NICU today, the faces I saw seemed totally new from what I had previously encountered. A new set of doctors had come on rotation, and there were nurses who had just returned from leave, as well as those who had switched from night duty to daytime duty. Some of the old doctors and nurses were still present though.

I located one of the doctors, D10 and started following her.

We were in Cubicle 2 seeing a very ill baby. The baby had been diagnosed with a congenital disorder, called hydrocephalus, which made the baby's head grow big at a fast rate.

He was alone in an incubator, and his father was often there to take care of me. The mum was unwell following childbirth, and is now just recuperating.

D10 wore her scrubs, a multi-colored top and plain trousers. She meticulously opened the incubator door and examined the baby. She had just finished seeing another baby when I walked in and she complained about the lack of running water on the unit. She picked up a bottle of alcohol and rub and realised it was empty. It did not surprise her, as she simultaneously lamented that these bottles are always empty.

She managed to find another bottle, which had some alcohol in it, and used it.

She examined the baby by putting the stethoscope on its chest.

She then stared at the baby's chest for a while, apparently counting the number of breaths.

She then unstrapped the baby's diaper and peeped in for a few seconds, checking for signs of ‘weewee’ and ‘poopoo’.

After she was done with the examination, she sat down to record her thoughts and findings in the baby's folder. I sat by her, and commented on her terrible handwriting, and we both laughed.

The desk for the doctor's is a wide desk with 2 chairs on either side, and an extra one or 2 chairs available for flexible use. A mother who was waiting for the doctor’s consultation sat with her baby in one of the chairs.

A while after D10 sat at the table, some other resident doctors approached her, asking questions on some babies they had reviewed. She appeared to have a lot of authority in the field.

There was a ward round ongoing in Cubicle 1, by one of the senior doctors. D10 was invited to join the ward round along with the other residents in the NICU.

I joined the ward rounds too.

The first patients we saw - 2 babies in a cubicle- I asked if they were related, but was told they were not related to each other - they were babies from different mothers

They were both described as ‘stable’, and there were discussions about discharging them home.

The next patient was a big baby, with very fast breathing, which they pointed out as ‘laboured breathing’.

There was a discussion as to why he was putting in so much effort to breathe, and the team leader suggested that they suction him, which they did.

They checked his drip which was hanging from a drip stand by the side of his court. The drip was labelled, with the amount of fluid and the period over which it had to run. The medical team noticed that he hadn't had enough fluids over the past 48hrs. The consultant(lead paediatrician) asked the house officers(interns) to correct the issue, and attend to the baby urgently.

The consultant then turned to the baby and said "Baby, forgive us our sins".

Then she went to wash her hands at the nearby sink which was outside the cubicle, and returned to continue the rounds.

The baby in the corner next to him had been brought from the maternity , with 2 different diagnosis. Discussions went back and forth as to which diagnosis was more likely. The team decided it was ‘Transient Tachypnea of the New born (TTN)’. There were discussions about the standard management protocols for this condition.

Another baby had been diagnosed of ‘meconium aspiration’, and had been started on antibiotics due to risk of sepsis.

We saw 2 babies sharing an incubator. One was very active, and seemed to be pushing away the other, who was rather quiet. One needed more warmth than the other, and there were discussions as to how to maintain a balanced optimal temperature in the incubator.

In the extreme right corner, 2 other babies shared incubator space. The oxygen had to be adjusted to meet the needs of both of them.

A very sick baby was seen toward the end of the ward rounds. The resident informed the consultant that the baby had not been reviewed by the night staff.

The consultant was displeased, and said that residents are supposed to review babies in order of the sickest baby- the one in the most critical condition, and not in order of how the babies were arranged in the cubicle during the ward rounds.

The last baby we saw had ‘Gastrochisis’, a condition in which the baby is born with bowels (abdomen contents) outside the abdomen. He had been managed by the paediatric surgeons, but the baby subsequently developed an infection. A bag hanging from the operation site was draining greenish fluid. One resident suggested that they changed the bag, but it meant the mother had to buy a new once, and funds would more efficiently be used for buying medicine. So, it was decided that the nurses should empty the bag , and re-use it.

There seemed to be a lot of knowledge at play through the course of the ward rounds. Residents were expected to know a lot, and the consultant demanded accountability for each baby. Someone was expected to be in charge, and that was the resident.

The house officers also have responsibilities, but they are lower in the hierarchy and reported to the residents. They would typically answer 'yes please' when speaking to the consultants, sometimes looking a bit intimidated.

The senior residents have a more cordial and interactive dialogue with the consultant.

| Health Facility code: FB |
| --- |
| Participants/Health worker code: Students/N3-6/D3-6/Other HPs |
| Unit name: NICU |
| Observer: MOK |
| Date: 24/11/2017 Observation Time: 9.30- 11.30am |

We had a focus group discussion with mothers in NICU.

Finding an appropriate space to conduct in-depth interviews with mothers remains a challenge.

On the NICU Ward, it’s a bit noisy with babies bursting into unpredictable bouts of crying and yelling.

The nurses also come round every now and then to administer drugs or care for the babies.

This interrupts the discussions.

It is also not practical to carry the mothers to a far away location: some of them are still tired and weak from surgery.

We found a side-room close to the NICU for the discussions.

THE USE OF GLOVES

The matron makes provision for items needed for each shift and leaves it in the care of the in charges. Any nurse who arrives first in each of the cubicle, takes the items needed for the shift. These items include a four packs of diapers, three tin of Pre Nan, three boxes of disposal gloves, a bottle of methylated spirit, one pack of cotton wool, dose flow and glucose strip. Each cubicle gets one box of disposal gloves which is used by doctors, nurses and rotation, student nurses.

In the morning, the gloves are usually enough by afternoon they run out of gloves and have to search for gloves in other cubicles. They pick gloves from the cubicles who have not run out yet and soon that cubicle also runs out. If they are unable to get the disposal gloves from other cubicles, they request for disposal gloves from the matron.

The nurses complain that when the student nurses come, they waste the gloves; they misuse gloves and tissues and soap. Some of the student nurses work with the gloves but others just wear the gloves without doing any work.

| Health Facility code: FA |
| --- |
| Participants: Mother, Shift nurses, Doctors |
| Unit name: NICU |
| Observer: GSM, MOK |
| Date: 20/12/2017 Observation Time: 9.00am-1.00pm |

9AM

Mothers who babies are in NICU are required to wash their hands with soap and water before entering the cubicle. Three sinks have been provided for the mothers, but two sinks are not functioning. The mothers have to depend on one sink which flows with water. Liquid soap and towels are provided beside the sink by the nurses. The mothers form one long queue to wash their hands because they have to depend on one tap which is working. The security woman instructs them to make the queue because at a point they were all rushing to the tap to wash their hand when it was time for them to enter NICU to feed their baby. As a result of the large numbers of the mothers, there was pressure on the mothers to wash their hands as fast as possible to give way for the next mother. Most of them did not wash their hand thoroughly.

12.00PM

Mothers were in a queue as usual, and one mothers seems to be taking long to wash her hand. Another mother from the back of the queue shouted at the mother washing hand. She said, *why is she taking so long?* *There are others in the queue waiting to wash their hands*.

There was a whole lot of confusion, the security woman had to come in and explain that the mothers have to wash their hands well.

After they wash their hands at the entrance, they walk into the cubicle take a plastic chair which they will be sitting on to feed their babies. They apply alcohol hand rub on they hands before picking up their babies. Some of them use the alcohol hand rub at the entrance of the cubicle before picking up the chair and feeding their babies.

| Health Facility code: FA |
| --- |
| Participants: N1, N2,N3, D1, D2 |
| Unit name: NICU |
| Observer: MOK, GSM |
| Date: 22/01/2019 Observation Time: 10.30-12.30pm |

I joined the staff in cubicle 3 to work today.

- I was assisting with drawing fluids and some medications for the babies.
- I also assisted with dispensing plaster for wound dressings and calming babies who were crying.
- I also run errands such as picking cannulas and syringes for the staff.

Observations:

1. Doctor setting an Intravenous line for a baby:

- In efforts to set lines for babies, it is quite difficult to find a vein and it takes quite a while.
- The doctors keep trying at various spots until the line is finally set.
- In between this, the gloves worn get stained with blood and there is usually no time to change these gloves.
- Doctors usually offer babies their fingers on which to suckling so as to soothe them. I observed how a doctor, in offering the baby a finger to soothe him, put a blood-stained gloved finger into the baby’s mouth. I drew the attention of the doctor and he tried to change the finger in question but did not change the glove which was stained.

1. Few Doctors on the ward, each working independently

- Since the doctors do not usually work in pairs when managing a baby, they tend to handle things with one hand while using the other hand on the baby.
- When there is the need to move to pick a thing or two, they have to leave the bedside and get the item and do not usually practice any hand hygiene when they return to take care of the baby

1. Activities of Student Nurses/ Rotation Nurses/ Interns

- Student nurses and nurses on rotation do not seem to have much knowledge about hand hygiene.
- I observed 2 of them moving from a baby to another without observing any form of hand hygiene. They rarely also wore the gloves.
- An interaction with them showed that they were not aware of the WHO five moments of hand hygiene, and a brief introduction was given.
- They were encouraged to practice hand hygiene as discussed

1. Emergencies

- There was an emergency where a baby was under respiratory distress. Some doctors were called in to aid in “bagging”.
- In the heat of the activities, very little hand hygiene was performed.
- Staff were seen moving around to pick an item or another that was needed to resuscitate the baby and returned only to start working on the baby without washing their hands nor using the hand sanitisers.

1. Generally, there was a lot of use of hand sanitisers among the staff than hand washing
2. The taps were flowing today; soap and towels were readily available at all the sinks.
3. The hand sanitiser dispensers still need some cleaning, and some were empty or near empty. The attention of the staff was drawn to that, and she agreed to have the student nurses take care of that.

| Health Facility code: FA |
| --- |
| Participants: Nurses/Other HPs/ Mothers |
| Unit name: NICU |
| Observer: EL |
| Date: 13/07/2018 Observation Time: 7.30- 12.30am |

NIKB-AB-18

Nurse arrived at 8:25 am, she wrote her name in the staff book to indicate she was present at work. She checked the assignment book to know which cubicle she is to work in. She was assigned to cubicle 3. The cubicle had a running tap, a diluted soap which was almost finished. She gathered the items she'll be needing for that shift. She directed the rotation nurses to take the vitals of the babies and also change the sheets and diapers.

At around 9:05, the mothers came in to feed. She wore a pair of gloves and took cups for mothers who had to express. She wore a new set pair of gloves and two fed a babies through an NG tube using the same gloves. She did not remove the pair of gloves or perform any hand hygiene in between.

She changed the baby’s bed sheet and placed the dirty sheet in the linen basket and diapers as well. She removed the gloves and stepped out of the cubicle without any hand hygiene. She took a cotton wool, dipped it into the bowl containing hot water to sterilize the cups which is used to feed the babies.

With the soaked cotton wool she wiped the baby’s nose which seemed to be filled with mucus. She dipped another cotton wool into the bowl and wiped the baby’s head to soften the plaster on the baby’s head to help it to remove easily. She wore gloves and passed feed through an NG tube for a baby. When it was 9:50 am she informed the mothers to change their baby’s diaper and burp them since it was almost time.

She cleaned the cot of a baby under the photometer and also changed the diapers. She directed a mother on where to get drugs and get her scan done.

After she wore a pair of gloves and fed the babies whose caretakers were absent . She didn’t perform any hand hygiene in between- only changed the gloves . She wrote some notes and came out of the cubicle and had a chat with the nurses at the reception table.

NIKB-SO-20

 Nurse arrived at 8:20 am, she wrote her name in the staff book to indicate she was present at work. She checked the assignment book to know which cubicle she is to work in. She was assigned to cubicle 1. She gathered the items she'll be needing for that shift.

A mother walked to her to give her breastmilk which she had expressed. She wore gloves on the one hand, leaving the other hand and fed the baby. She then fixed the NG tube into the baby’s nose and removed the gloves. No hand hygiene was performed after, she touched another baby without wearing gloves.

She later fed a baby under the Radiant warmer through an NG tube and tapped the baby’s back to burp. She went ahead to chart the feed.

She administered another baby’s medication and regulated the baby’s monitor. After, she checked the vitals of a baby in an incubator and recorded it in the folder. She checked the vitals of three more babies in an incubator without any hand hygiene in between or gloves. After checking the vitals she used alcohol hand rub and returned to one of the babies in an incubator. She cleaned the table with cotton wool and methylated spirit.

At 12:15 pm, the mothers came in to feed. When the mothers arrived she handed cups to them and stepped out of the cubicle to wash her hand.

p

| Health Facility code: FA |
| --- |
| Participants: Students/N3-6/D3-6/Other HPs |
| Unit name: NICU |
| Observer: MOK |
| Date: 28/01/2019 Observation Time: 9.30- 11.30am |

There were a lot of students today at the NICU.

For all but cubicle one, there was only one staff nurse and several students (6-10).

The nurses in the various cubicles doubled as nurses for the babies and also as tutors for the students.

Intermittently they would stop and teach the students on things that need to be done.

I noticed that some of the students had gloves on even when they were doing nothing.

These gloves are not taken off nor changed in the course of time.

For the staff (doctors and nurses), they appeared conscious of my presence and tried hard to observe the hand hygiene especially whenever they noticed me looking on. Occasionally a would pass a comment to suggest their awareness of my presence (hygiene police, hand hygiene, is eating against IPC rules?, etc).The students seemed oblivious of my observations and were more relaxed where hand hygiene was concerned.

One nurse had to stop feeding a baby to help a student clean up an incubator. They used a liquid solution of parazone and a small towel to wipe the incubator clean after removing the mattress and the moveable parts of the incubator. According to the nurse in that cubicle, it is occupied by a baby who has an infection that is not resolving. The baby has been prescribed some systemic antibiotics. The baby was at the time being taken through KMC(Kangaroo Mother Care) and the nurse had asked a student to clean the cubicle while the baby is away.

I noticed also that although the alcohol hand rubs were available, the mothers rarely used them. Many of them (about 20 mothers in C1) after washing their hands at the entrance went through their routines without performing any hand hygiene. Some mothers (3)were seen using the wipes to clean their hands when they thought it was important to have them cleaned.

I also noticed how staff with their gloved hands, operated certain equipment and with that same hand worked on the babies. In an attempt to revive a baby, a nurse, with her hands gloved, kept adjusting some functions on the cot in which the baby was lying. After this, she again started working on this baby without changing the gloves or performing any other hand hygiene. This was repeated by a doctor on the same baby.

The sinks in the cubicles are working and have a good stock of soap, alcohol hand rub and towels. The taps were flowing too.

Staff were more attentive to performing hand hygiene before they approached a baby but in between procedures, whenever they have to move to pick an item or do something before returning to the babies, hand hygiene is mostly not performed.
